# Combination treatment with U0126 and rt-PA prevents adverse effects of the delayed rt-PA treatment after acute ischemic stroke

**DOI:** 10.1038/s41598-021-91469-9

**Published:** 2021-06-07

**Authors:** Cyrille Orset, Kajsa Arkelius, Antoine Anfray, Karin Warfvinge, Denis Vivien, Saema Ansar

**Affiliations:** 1 INSERM UMR-S U1237, Physiopathology and Imaging of Neurological Disorders, GIP Cyceron, Institut Blood and Brain @ Caen-Normandie (BB@C), Bd H. Becquerel, BP 5229, 14074 Caen, France; 2 Applied Neurovascular Research, Neurosurgery, Department of Clinical Sciences, Lund University, Klinikgatan 28, BMC C12, 222 42 Lund, Sweden; 3 Experimental Vascular Research, Department of Clinical Sciences, Lund University, Lund, Sweden; 4 Department of Clinical Research, Caen-Normandie University Hospital, CHU, 14000 Caen, France

**Keywords:** Drug discovery, Molecular biology, Neuroscience, Diseases, Neurology

## Abstract

In acute ischemic stroke, the only FDA-approved drug; recombinant tissue plasminogen activator (rt-PA) is limited by restricted time-window due to an enhanced risk of hemorrhagic transformation which is thought to be caused by metalloproteinase (MMP). In experimental stroke inhibitors of the mitogen–activated protein kinase kinase extracellular signal–regulated kinase kinase (MEK) 1/2 pathways reduce the MMPs. This study evaluated whether a MEK1/2 inhibitor in combination with rt-PA can prevent the detrimental effects of delayed rt-PA therapy in stroke. Thromboembolic stroke was induced in C57 black/6J mice and the MEK1/2 inhibitor U0126 was administrated 3.5 h and rt-PA 4 h post stroke-onset. Treatment with rt-PA demonstrated enhanced MMP-9 protein levels and hemorrhagic transformation which was prevented when U0126 was given in conjunction with rt-PA. By blocking the MMP-9 with U0126 the safety of rt-PA administration was improved and demonstrates a promising adjuvant strategy to reduce the harmful effects of delayed rt-PA treatment in acute ischemic stroke.

## Introduction

Ischemic stroke remains a leading cause of death and disability worldwide and is an enormous economic, clinical and social burden. Current treatments for acute ischemic stroke are limited to thrombolytic therapies involving the use of endovascular thrombectomy, and the only effective FDA approved drug recombinant tissue plasminogen activator (rt-PA)^1^. Although rt-PA represents an important break-through for management of acute stroke, this treatment has several limitations, recanalization efficiency is far from optimal, and it can only be administered within 4.5 h after stroke onset because of the risk of hemorrhagic transformation^2,3^, cerebral edema and neurotoxicity^4^. Less than 10% of all stroke patients receive rt-PA, because of the narrow time-window^5^. The hemorrhagic transformation can lead to significant morbidity and mortality in stroke patients. It is thought that metalloproteinases (MMPs) are the key culprit in causing hemorrhagic transformation. Elevated plasma levels of MMP are correlated with the frequency of hemorrhagic transformation following stroke^6,7^*.* MMP precursors are expressed in brain blood vessels within several hours after ischemia, and these precursors become active enzymes if cleaved by rt-PA and its substrate plasmin, leading to breakdown of extracellular matrix and leakage in the vessel; this mechanism is thought to underlie hemorrhagic transformation. When a brain vessel is occluded, the reduction in blood flow triggers molecular regulatory “switches” that turn on a vascular injury response program that includes MMP expression. The mitogen-activated protein kinase kinase extracellular signal-regulated kinase kinase (MEK) 1/2 signaling pathway is an important “switch” in the blood vessel wall that leads to upregulation of MMP-9 as a response to ischemia. Studies have previously demonstrated that inhibitors targeting MEK1/2 reduces vascular MMP levels following experimental stroke^8,9^. By blocking the MMP-9 by adding a MEK1/2 inhibitor in combination with rt-PA, we propose that we can improve the safety of rt-PA administration by preventing the hemorrhagic transformation. Here, we investigated whether the combination therapy of the MEK/1/2 inhibitor U0126 and rt-PA can alleviate the detrimental effects of delayed rt-PA therapy in acute ischemic stroke.

## Results

### U0126 prevent the deleterious effects of delayed thrombolysis by rt-PA

**Figure 1:**
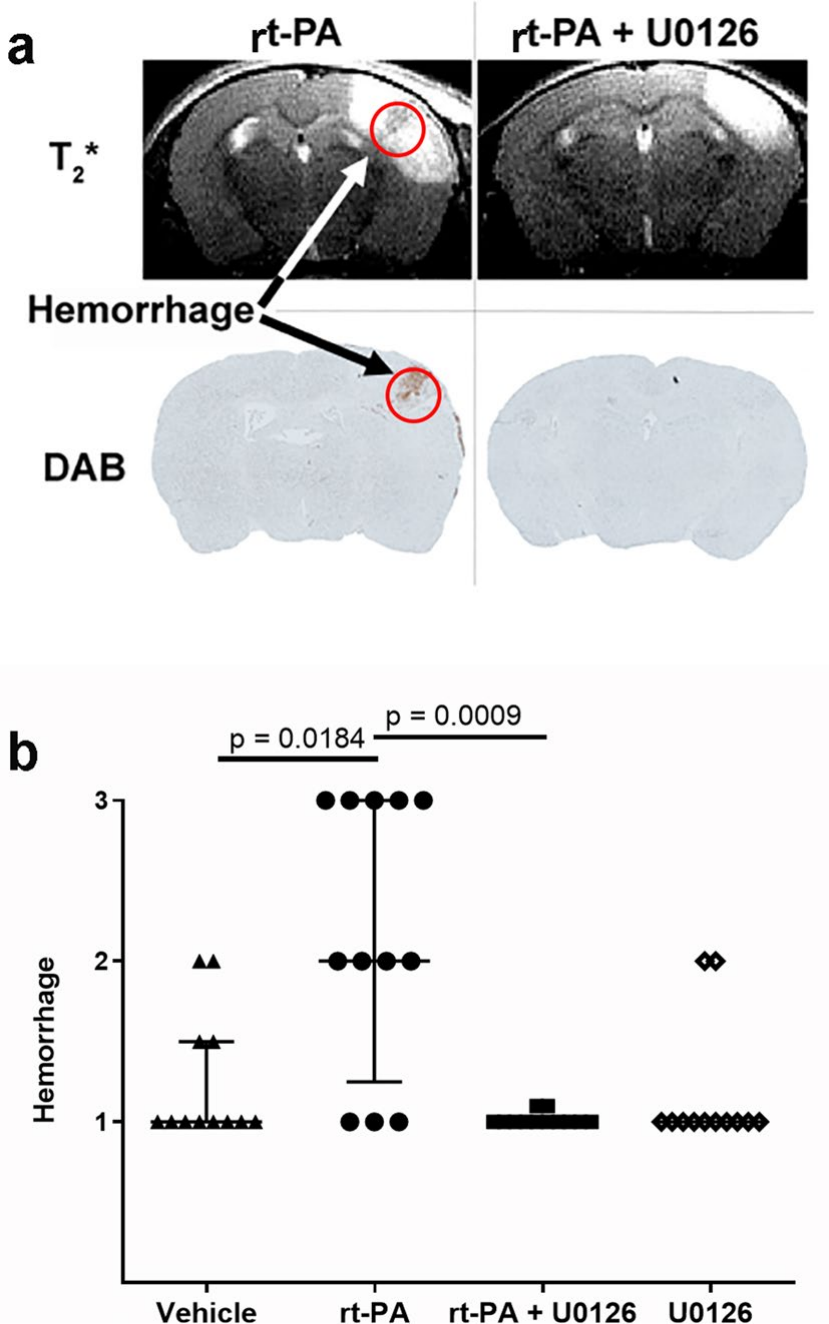
(**a**) Representative T_2_*-weighted images and diaminobenzidine staining of the same slice showing hemorrhages marked by arrows. (**b**) Combination treatment with rt-PA + U0126 prevented the rt-PA induced hemorrhagic transformation. Quantification of hemorrhages were evaluated using DAB staining. Data are presented as median ± interquartile range (IQR), *P < 0.05 is considered statistically significant.

**Figure 2:**
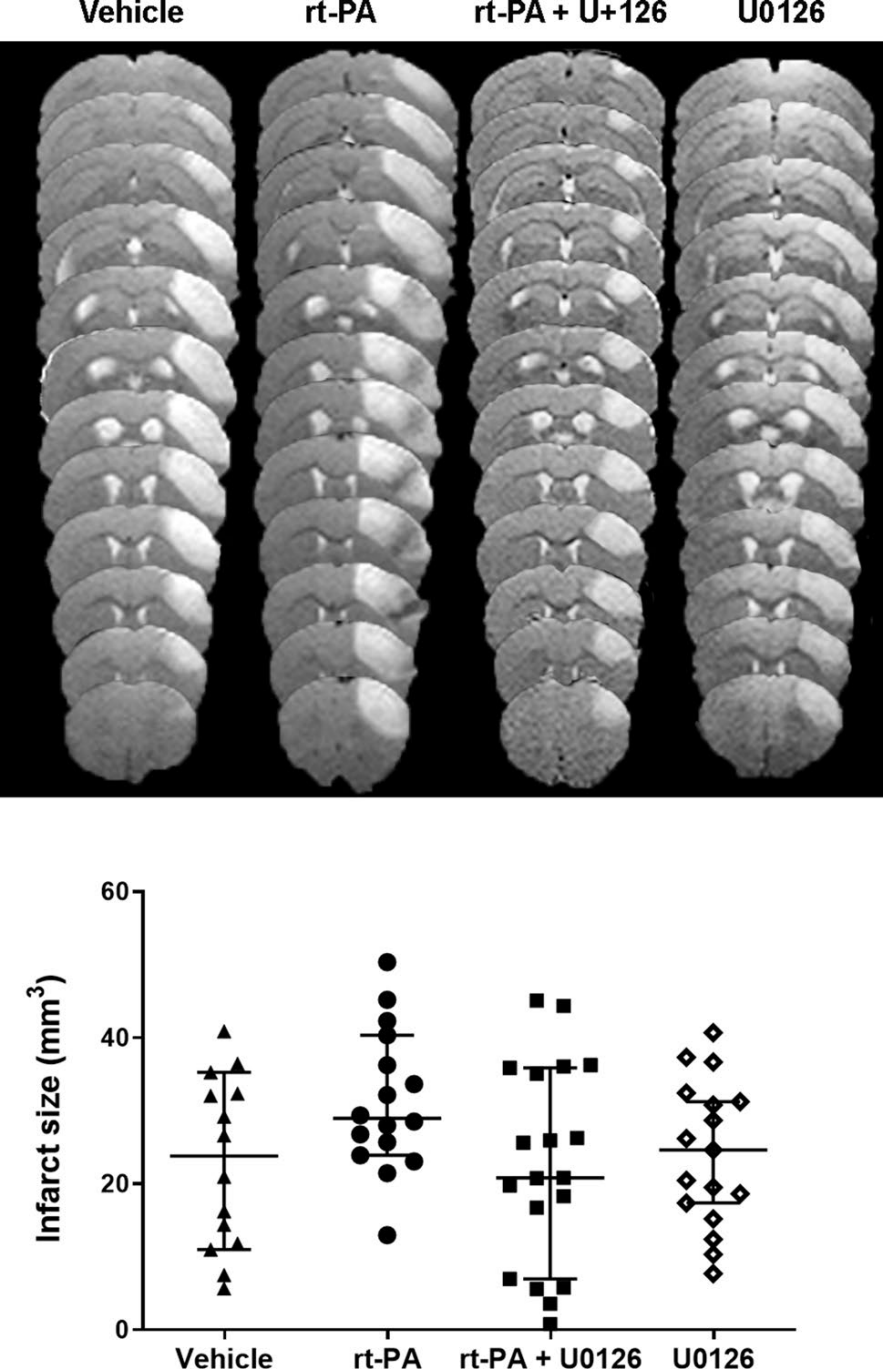
Combination treatment with rt-PA + U0126 did not affect the infarct size after stroke compared to rt-PA and vehicle group. Representative T2-weighted image visualizing the infarct lesion within the cerebral cortex of the different treatment groups. Data are presented as median ± IQR, *P < 0.05 is considered statistically significant.

Thrombin injection stabilized clot formation and induced cortical brain injury in all animals included in the study (n = 64). Delayed treatment with rt-PA was associated with worsened hemorrhagic risk (Fig. 1a,b). Hemorrhages were more frequent and more severe in the rt-PA treated animals compared to vehicle group (2.00 (1.25, 3.00), respectively 1.00 (1.00, 1.50), p = 0.0184). Combination therapy with rt-PA and U0126 significantly prevented the rt-PA induced hemorrhagic transformation (1.00, (1.00, 1.00), p = 0.0009) (Fig. 1a,b). However, there was no difference in infarct size between the groups, vehicle: 23.79 (11.61, 33.07) mm^3^, rt-PA: 28.95 (24.34, 39.31) mm^3^, rt-PA + U0126: 20.82 (6.98, 35.86) mm^3^, U0126: 24.62 (16.29, 31.83) mm^3^ (Fig. 2). Infarct volume was measured 24 h following thromboembolic stroke by MRI.

### U0126 and rt-PA in combination prevent activation of MMP-9

**Figure 3:**
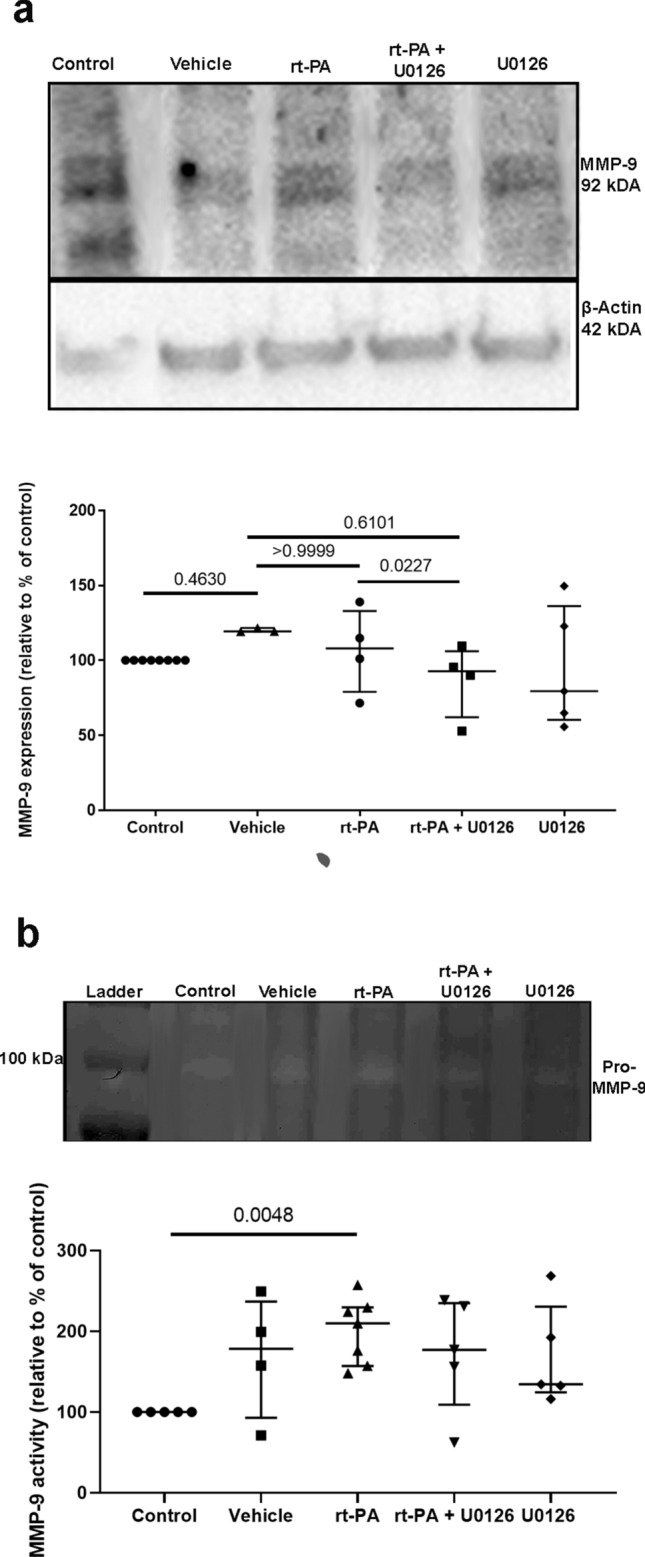
Combination treatment with rt-PA + U0126 prevents the increased MMP-9 expression after delayed rt-PA treatment. (**a**) Representative western blots and scatter plots displaying expression of MMP-9.MMP-9 expression levels were normalized to control. (**b**) Representative zymography blots and scatter plots illustrate MMP-9 protein expression. The expression levels were normalized to control group which was set to 100%. All data are presented as median ± IQR, *P < 0.05 is considered statistically significant, n = 3–7.

**Figure 4:**
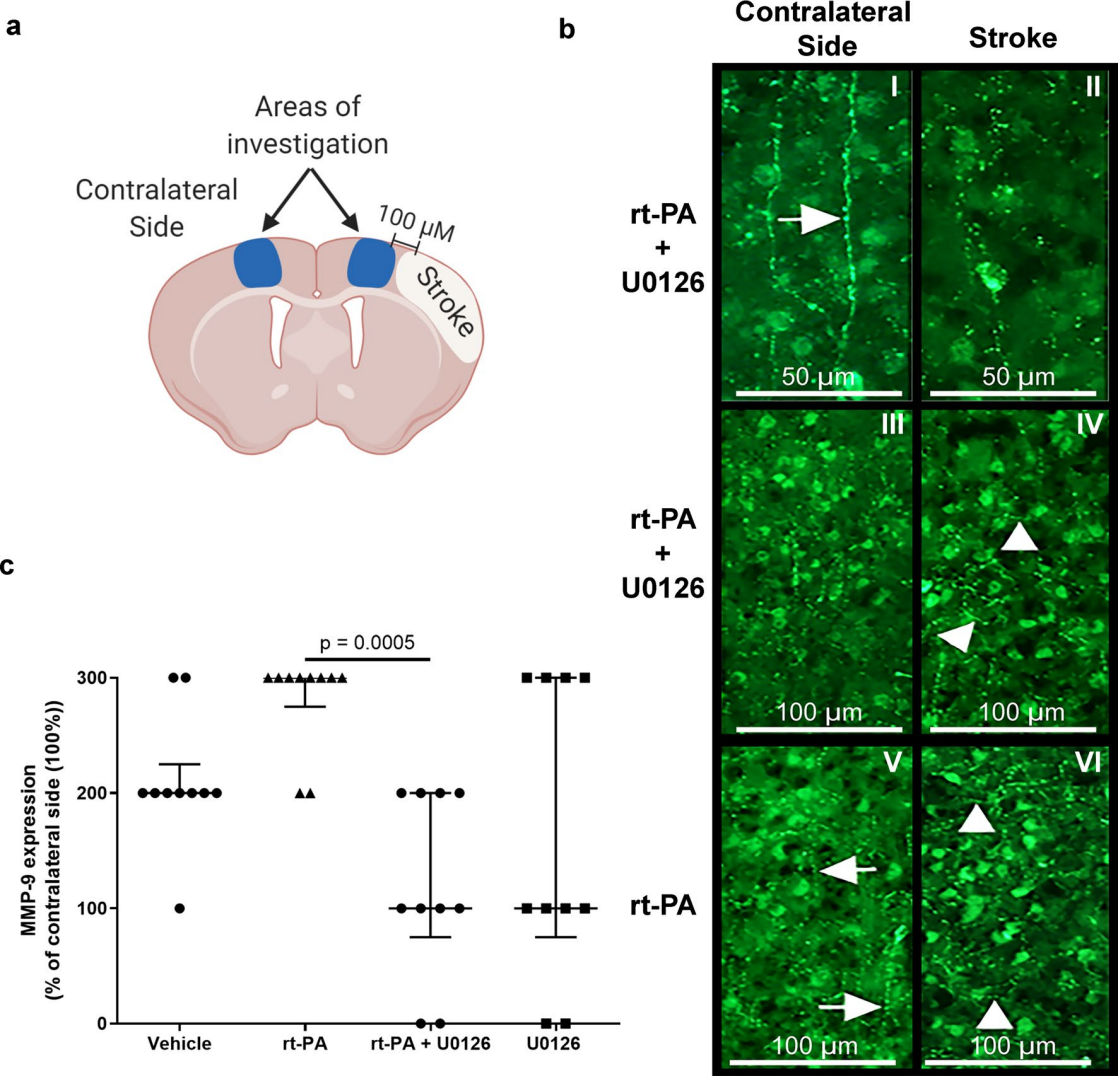
Representative images of MMP-9 immunoreactivity. (**a**) Illustrationimmunoreactivity images for MMP-9. All images were obtained in cortical layers III–V, 100 µm towards the midline in relation to the stroke area. Created with BioRender.com (https://biorender.com) (**b**) U0126 + rt-PA 50 µm: I. The immunoreactivity in the brains control side was organized in the radial orderliness of the cortical neurons. Arrow points at pearl-like MMP-9 immunoreactivity. II. The corresponding area in the stroke side of the same individual showed less defined structural organization. U0126 + rt-PA 100 µm: III. The image demonstrates a lower magnification of the control side of the brain. IV. In the stroke side of the individual in III, it became obvious that, apart from the radial organization, the immunoreactivity was organized in a more or less perpendicular to the cortical neuronal format (arrow heads), which was not often found in the control side. rt-PA V. In the control side of the animals treated with rt-PA, the immunoreactivity displayed similar features as for U0126 treated animals shown in I and III, with often radial distribution of the immunoreactivity (arrows). VI. In the stroke area of the animal shown in **V**, a disorganization of the MMP-9 immunoreactivity was clearly apparent (arrow heads). (**c**) Scatter plot showing semi-quantification of protein expression for MMP-9. Data are presented as median ± IQRand normalized to control. *P < 0.05.

Zymography and western blot analysis were performed to investigate the expression of MMP-9 protein levels. We observed that MMP-9 protein levels were significantly decreased when U0126 was combined with rt-PA compared with rt-PA treatment alone. However, MMP-9 activity was not different between the groups (Fig. 3, Supplementary Fig. 1). Similar results were observed with the immunohistochemistry (Fig. 4). Using NIS basic research software, the ischemic area in each section was delineated as shown in Fig. 4a and the overall MMP-9 immunoreactivity was examined. The immunoreactivity in the control side of the brain was often organized in a radial pattern, corresponding to the distribution of the cortical neurons (Fig. 4b). Often a pearl-like distribution was observed (Fig. 4b-I), suggesting staining of the neuronal processes. The distribution of the immunoreactive structures in the stroke areas was different in all groups (Fig. 4b). In addition to the radial distribution observed in the control side, a transverse distribution was found. This disorganization was found in all stroke sides, however, more or less pronounced. The amount of immunoreactive structures increased, with the largest difference observed in the group treated with rt-PA (Fig. 4c).

### U0126 and rt-PA in combination prevent activation of pERK1/2

**Figure 5:**
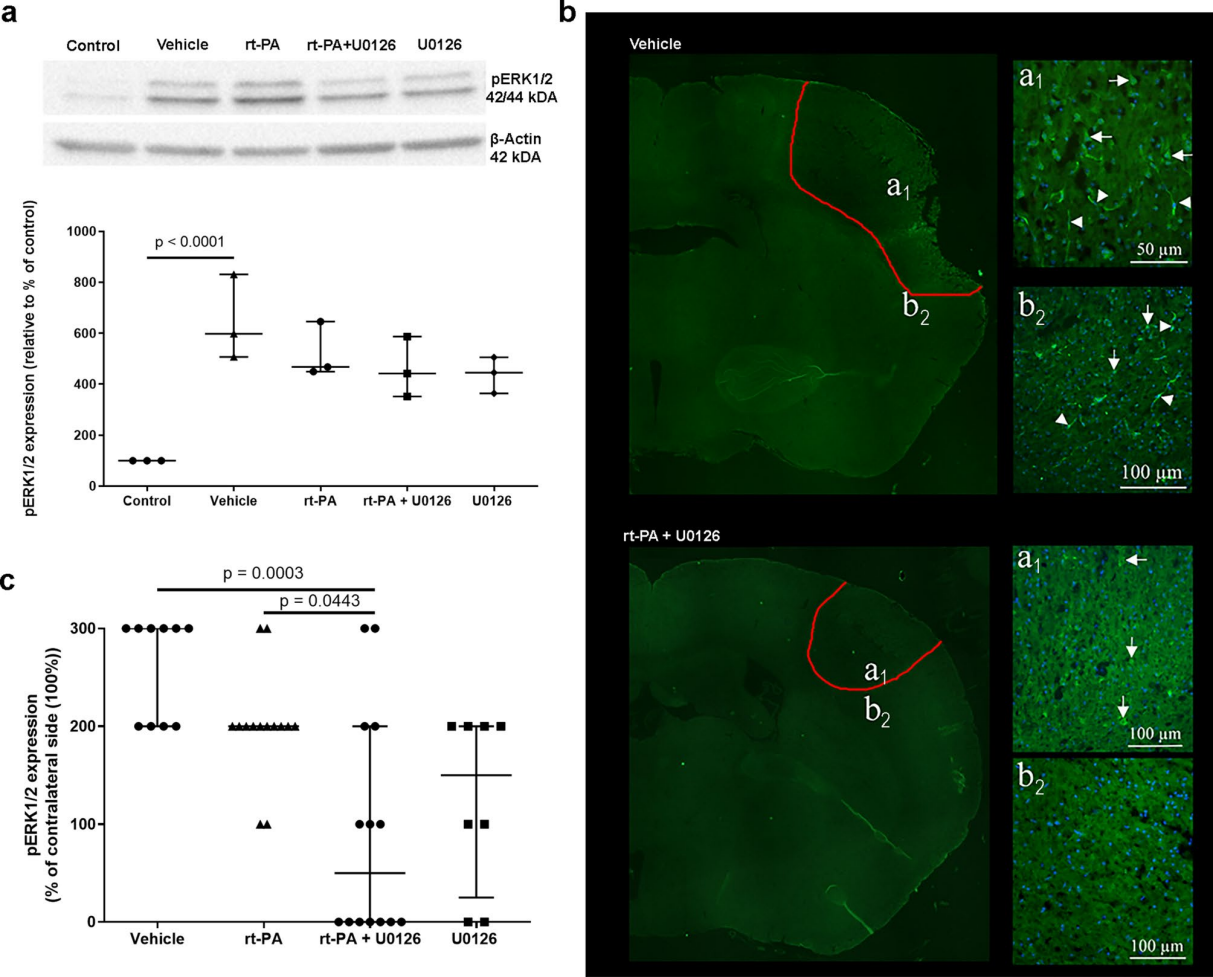
(**a**) p-ERK1/2 protein expression is shown by representative western blots and scatter plots. The protein expression of p-ERK1/2 was normalized to β-actin (loading control). In all comparisons, the average value for the control group was set to 100%. p-ERK1/2 has a molecular weight of 42/44 kDa and the one of β-actin is 43 kDa. Data are presented as median ± IQR, *P < 0.05 is considered statistically significant. (**b**) Representative image of pERK1/2 immunoreactivity. Upper panel, saline treated animal. To the left, area of stroke (outlined in red) is illustrated in a large image. Letters a_1_ and b_2_ refer to the illustrations to the right. a_1_. The stroke core is shown. Arrows point at pERK1/2 immunoreactive cells and arrow heads at vessels. b_2_. Immunoreactive cells and vessels were also found close to the stroke area. Lower panel, t-PA + U0126 treated animal. To the left, red-outlined stroke areas is illustrated in a large image. Letters a_1_ and b_2_ refer to the illustrations to the right. a_1_. Some pERK1/2 immunoreactive cells (arrows) were found in the stroke core, but not in the area outside the stroke b_2_. (**c**) Scatter plot showing semi-quantification of protein expression for pERK1/2. Data are presented as median ± IQR, and normalized to the non-occluded side. *P < 0.05.

The pERK1/2 protein levels were significantly increased after ischemia (Fig. 5a). Interestingly, there was no effect of rt-PA treatment, nevertheless rt-PA in combination with U0126 and U0126 alone showed a tendency to prevent the enhanced protein level observed after ischemia (Fig. 5a), though no differences were observed. Similarly, there was an increase in pERK1/2 immunoreactivity after ischemia (Fig. 5b,c). Both combination treatment with rt-PA and U0126 and U0126 as a single treatment prevented this increase (Fig. 5c). Immunoreactivity of pERK1/2 was only found within the stroke core and in areas close to stroke in all groups. Consequently, only these areas are described below. All animals in the vehicle group contained immunoreactive cells in the core and the area close to the core. Also, immunoreactive vessels were found (Arrow heads; Figs. 5b). In the other groups, the number of positive cells decreased, with fewest immunoreactive cells in the group treated with the combination therapy rt-PA + U0126. In this group, no positive cells were found in the area close to the stroke area (Fig. 5b). In the groups treated with U0126, or rt-PA in combination with U0126, no immunoreactive vessels were found.

## Discussion

This study evaluated the important question whether rt-PA-associated hemorrhage after delayed rt-PA treatment in an animal model of thromboembolic stroke can be prevented by blocking the MMP-9 through a specific MEK1/2 inhibitor. Our data demonstrate that by blocking the MMP-9 expression through the use of a MEK1/2 inhibitor the rt-PA induced hemorrhagic transformation was prevented, indicating that this may be a promising adjuvant strategy to alleviate the detrimental side effects of delayed rt-PA in acute ischemic stroke. However, there was a discrepancy between the effect on the cerebral infarct volume and hemorrhagic transformation. No effect on the infarct volume was observed even if the hemorrhagic transformation was prevented. In previous study where U0126 was administrated at both 6 h and 24 h after tMCAO a reduced infarct compared to vehicle was demonstrated over time with a significant difference at day 14 post-stroke^10^, but not at early time-points. The same dose was used in this study 30 mg/kg, however as one single dose, which may be one of the reasons why U0126 alone did not have any protective effect in the current study. Further explanations may be that the timepoint 24 h was too early to evaluate the infarct and that we still have ongoing processes and changes in infarct may occur much later. The activation of MMP-9 and enhanced expression of pERK1/2 are expressed in the vasculature. The MMP precursors are expressed in brain blood vessels after ischemia, and become active enzymes if cleaved by rt-PA, leading to vascular dysfunction, and hemorrhage resulting in neuronal damage. By preventing the hemorrhagic transformation the neuronal damage may become reversible which can be observed over time. Further long-term studies will be needed to assess the long-term outcome. The only effective drug therapy for treatment of acute ischemic stroke to date is thrombolysis with rt-PA^1^. The major complication of rt-PA is the hemorrhagic transformation which is associated with increased stroke morbidity and mortality. Recombinant tissue plasminogen activator increase the rate of hemorrhage by tenfold^11,12^ and given that rt-PA is the only FDA approved treatment for acute ischemic stroke it is essential to prevent the detrimental effects of rt-PA and restore blood flow without complications. Elevated MMP plasma levels have been reported to correlate with the frequency of rt-PA induced hemorrhagic transformation following stroke in both animal models^6,7^ and stroke patients^13^. It is demonstrated that rt-PA activates nuclear factor (NF)-КB signalling leading to activation of MMP-9, which in turn contribute to enhanced blood–brain barrier (BBB) permeability, vascular dysfunction and hemorrhage^14,15^. Despite the correlation between enhanced MMP-9 levels after rt-PA treatment and worsen outcomes of patients, there is no current therapy to prevent hemorrhagic complications. Our present study demonstrates that the specific MEK1/2 inhibitor U0126 prevent activation of MMP-9 in the acute phase of experimental stroke, which is in agreement with a previous study^16^. By adding U0126 in combination with rt-PA we prevent activation of MMP-9 expression leading to BBB leakage and hemorrhagic transformation. Despite tremendous attempts over the last decades in developing new treatments for ischemic stroke none of them demonstrated efficacy and safety in clinical trials^17,18^. Considering that stroke is a vascular disease, neuroprotection without restoration of tissue perfusion will have a low possibility of demonstrating efficiency in treating acute ischemic stroke^19^. In the present study, we use rt-PA to restore the cerebral blood flow and by adding U0126 as an adjuvant agent to block MMP-9 we are able to prevent the enhanced MMP-9 leading to BBB breakdown and hemorrhagic transformation which is one of the major complications of delayed rt-PA treatment. In experimental ischemic stroke models broad spectrum MMP inhibitors have shown reduced infract and BBB permeability, however, clinical trials resulted in unsatisfactory outcome due to toxicity and adverse effects. It has been technically challenging to develop specific MMP-9 inhibitors, but recent generation of MMP inhibitors have overcame this obstacle and shown improved pharmacokinetics^20^. In this study we demonstrated that by indirectly inhibiting MMP-9 the hemorrhagic transformation was prevented. This suggest that targeting the MMP-9 activation may be a promising strategy to prevent the detrimental side effects of delayed rt-PA therapy. However, further studies need to be performed to investigate the use of the new promising MMP-9 inhibitors in acute ischemic stroke as well as functional and long-term outcome.

## Materials and methods

### Experimental design of the study

**Figure 6:**
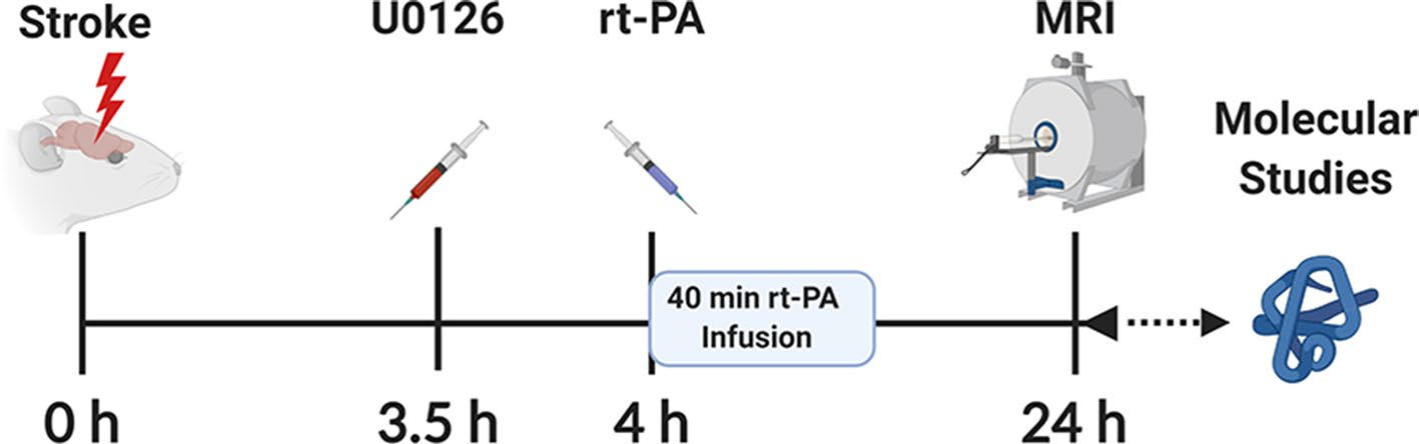
Illustration of the study design is created with BioRender.com. (https://biorender.com).

Thromboembolic stroke was induced in C57BL/6J mice (20–30 g) and all animals were randomly divided into 4 groups; (i) vehicle treated, (ii) treated with rt-PA, (iii) treated with rt-PA in combination with U0126 and (iv) treated with U0126. To induce thrombolysis, rt-PA (10 mg/kg; Actilyse) was intravenously administrated 4 h after thrombin injection. It was injected as 10% bolus and 90% perfusion for 40 min. The MEK1/2 inhibitor U0126 (30 mg/kg; LC laboratories, Boston, MA, USA) or vehicle (dimethyl sulfoxide) was injected intraperitoneal at 30 min prior to rt-PA administration, in a random and blinded fashion. The dosage for U0126 was chosen based on previous studies^21^. After 24 h all mice were euthanized and brains removed and either fixed in 4% paraformaldehyde in phosphate buffered-saline (PBS) for immunohistochemistry and diaminobenzidine (DAB) staining or snap frozen in isopentane in order to collect tissue for western blot and zymography. The design of the study is illustrated in Fig. 6.

### Ethics approval

All experiments were carried out in strict accordance with the guidelines for the European Community Council Directive (2010/63/EU) for Protection of Vertebrate Animals Used for Experimental and other Scientific Purposes and were approved by the Malmö-Lund Institutional Ethics Committee under the Swedish National Department of Agriculture (Animal Inspectorate License No.M86-15 and French ethical laws (act no. R214; 87–137 du code rural) and approved by the French ethical committee (under the identification number CENOMEXA 0113–03). The study complies with the ARRIVE guidelines (Animal Research: Reporting In Vivo Experiments).

### Thromboembolic stroke

Thromboembolic stroke was induced by local injection of thrombin directly into the right middle cerebral artery (MCA) of mice as originally described by Orset et al.^22^. Male C57 black/6J mice (20–30 g) were anaesthetized using 5% isoflurane and thereafter maintained with 1–2% isoflurane during the surgical procedure. An electric temperature probe was inserted into the rectum of the mouse to record the temperature, and found to be maintained at 37 °C. A catheter was placed in the tail vein to allow the intravenous administration (200 µl) of vehicle or t-PA. The mice were placed in a stereotaxic device and the temporal muscle was retracted. A small craniotomy was performed, the dura was excised, and the MCA was exposed. The laser Doppler flow probe to measure cortical cerebral blood flow (CBF) was placed on the skull in the MCA territory. Finally, a micropipette filled with 1 µl of purified murine alpha-thrombin (1.5 UI) was introduced into MCA bifurcation lumen and injected carefully to induce the formation of a clot in situ*.* The pipette was removed 10 min after the injection at which time the clot had stabilized. CBF velocity was measured continuously by laser Doppler flowmetry allowing determination of spontaneous clot dissolution. A clot was defined as successful when the cerebral blood velocity decreased minimum 60% from baseline at the time of thrombin injection and remained stable during minimum 20 min.

### Magnetic resonance imaging

All experiments were performed on a Pharmascan 7T (Bruker, Germany) small animals’ system. Animals were anaesthetized with 5% isoflurane and thereafter maintained with 1–2% isoflurane. During the scanning procedure body temperature was maintained using a water-heating system and breathing rate was monitored. The MR data set was acquired at 24 h after the onset of thromboembolic stroke. T2-weighted images to visualize infarction were acquired using MSME sequences (multi-spin multi-echo): echo time/repetition time: 51 ms/2500 ms with a 70 mm × 70 mm × 500 mm spatial resolution. Lesion size was measured 24 h post-surgery on T2-weighted images using ImageJ software. The measurements were performed by a blinded evaluator.

### Intracerebral hemorrhage examination

To evaluate the presence of hemorrhage after delayed rt-PA treatment, T2^*^-weighted gradient echo images by MRI and diaminobenzidine staining was performed on 10 µm coronal cryosections. Diaminobenzidine staining is commonly used to define hemorrhagic areas in sections^23^ and has previously been described^24^ Hemorrhages were assessed by blinded histological evaluation on three sections from each animal. The incidence of hemorrhagic transformation was determined and counted microscopically by classifying them into grades 1–3; 1 = no hemorrhage, 2 = defined as more confluent petechiae within the damaged area, 3 = defined as blood clots in more than 30% of the damaged area.

### Tissue collection for Zymography and western blot

Brains frozen in isopentane were used to collect tissue for zymography and western blot. Tissue samples were collected from the ischemic and contra-lateral side of each brain. All tissue samples were collected blinded. Tissues collected for all four groups were further used for protein extraction. Total protein concentration was determined using a BioRad Protein Assay kit (Hercules, CA, USA).

### Zymography

Lysates from each sample (20 µg protein) was mixed with Tris–Glycine SDS Sample Buffer (2X) (1:1) and separated at 4 °C on 10% Tris–Glycine SDS-PAGE (0.1% Gelatin) protein gel (Thermo Fisher Scientific Life Technologies, Carlsbad, CA, USA). A molecular weight marker (PageRuler Plus) was loaded on each gel for protein band identification. The gel was then incubated at room temperature for 30 min with a zymogram renaturing buffer before incubation for 3.5 days at 37 °C in a zymogram developing buffer. Bands were visualized by Coomassie staining.

### Western blot

To evaluate the protein levels western blot was used^25^. Briefly, equal amounts of protein (50 µg) samples were mixed with Laemmli Sample Buffer, boiled for 4 min and loaded onto a 4–20% mini-protean TGX gel (Bio-Rad laboratories, CA, U.S.A.) and separated by SDS-PAGE. Molecular weight marker (Precision Plus Protein Kaleidoscope, Bio-Rad laboratories, U.S.A.) was loaded on each gel for protein band identification. After gel electrophoresis the proteins were transferred to a nitrocellulose membrane and incubated overnight with primary antibody; MMP-9 (1:400, Santa Cruz Biotechnology, Dallas, TX, U.S.A) or phospho-ERK p44/42 MAPK (1:2000, Cell Signaling Technology, Beverly, CA, U.S.A., #9101) at 4 °C. Subsequently, the membranes were incubated with the secondary antibody; anti-rabbit for phosphor-ERK p44/42 MAPK (1:2000, Cell Signaling Technology, Beverly, CA, U.S.A., #7074), and anti-goat for MMP-9 (1:4000, Santa Cruz Biotechnology, Dallas, TX, U.S.A, sc-2033) for 1 h at room temperature. The membranes were developed using the Supersignal Dura kit and protein bands were visualized using Fujifilm LAS1000 image analyzer. Subsequently the membranes were stripped and reprobed with β-actin (1:50,000, SIGMA-ALDRICH, Darmstadt, Germany, A3854). Three independent experiments were performed for each primary antibody.

### Zymography and western blot analysis

Protein lysates from the 4 different groups and samples from the right hemisphere of the mice were compared. Quantification of band intensity was performed by using image J software (http://rsb.info.nih.gov/ij/). The optical density values were determined with repeated measurements and presented as percentage activity of the treated group compared with the control group (tissue from healthy mice), where the values for the control group were set to 100%. Each sample was replicated three times. For western blot the intensity of the bands for pERK1/2 and MMP-9 was normalized to β-actin which was used as loading control.

### Immunohistochemistry

Immunohistochemistry was performed as previously described^10^. Brains were fixed in 4% paraformaldehyde and then sectioned into 12-μm thick slices in a cryostat. Brain sections were permeabilized in PBS containing 0.25% Triton X-100 (PBST) and blocked for 1 h in blocking solution containing PBS 0.25% Triton X-100, 1% BSA and 5% normal goat serum. The sections were incubated over night at 4 °C with the following primary antibodies: rabbit monoclonal MMP-9 (ab137867, Abcam, Cambridge, UK) diluted 1:100 or mouse dually phosphorylated ERK1/2 (#ab50011, Abcam, Cambridge, UK) diluted 1:200. The sections were subsequently incubated for 1 h at room temperature with FITC-conjugated donkey anti-mouse (1:100, Jackson ImmunoResearch) or FITC-conjugated goat anti-rabbit (1:100, Jackson ImmunoResearch) as secondary antibody. The sections were mounted with a mounting medium containing nucleus staining DAPI (Vecta-Shield, Vector Laboratories, Burlingame, CA, U.S.A.). The experiments were repeated to ensure reproducibility. Negative control was performed by omitting primary antibody. Brain sections were chosen where the infarct size was at largest, accordingly to the MRI measurements. Immunoreactivity of the brain sections was visualized with an epifluorescence microscope (Nikon 80i; Nikon, Tokyo, Japan) coupled to a Nikon DS-2 MV camera at the appropriate wavelength. All the images were taken at the same day for all the sections and images analysis were performed blinded. The image was taken on each of four sections for all the individual mice.

### MMP-9

Using NIS basic research software, the ischemic area in each section was delineated and the overall MMP9 immunoreactivity was examined. 100 µm towards the midline in relation to the ischemic area, an area measuring approximately 300 µm in width and spanning through all cortical layers was outlined. The corresponding area in the control side was defined and examined. The immunoreactivity here was judged as a base-line regarding the particular individual. A three-grade scale was used to examine the intensity, distribution and the structural features of MMP9 immunoreactivity in the ischemic area in relation to the base-line in the control side.

### pERK1/2

The ischemic area was delineated and the overall pERK1/2 immunoreactivity was examined. As immunoreactivity was observed specifically within the stroke area and areas close to stroke, the calculations were performed in these. A three-grade scale was used to examine the intensity and distribution of pERK1/2 immunoreactivity.

### Statistical analysis

Data are expressed as median ± interquartile range (IQR). P < 0.05 was considered as significant and “n” refers to the number of mice. Kruskal–Wallis test with Dunns multiple comparison analysis was performed by using GraphPad Prism 7.03.

## Supplementary Information

Supplementary Information.

## Data Availability

The data that support the findings of this study are available from the corresponding author upon reasonable request.
