# Supplementary material for: Combination treatment with U0126 and rt-PA prevents adverse effects of the delayed rt-PA treatment after acute ischemic stroke
Source: Sci Rep. 2021 Jun 7;11:11993. doi: 10.1038/s41598-021-91469-9 (PMC8184783; doi:10.1038/s41598-021-91469-9)
Supplement: Supplementary file 1 — Supplementary Information. [file 41598_2021_91469_MOESM1_ESM.pdf]

Supplemental Material

Combination treatment with U0126 and rt-PA prevents adverse effects of rt-PA therapy after acute ischemic stroke

Cyrille Orset[1], PhD; Kajsa Arkelius[2], MSc; Antoine Anfray[1], PhD; Karin Warfvinge[3], DD PhD; Denis Vivien[1,4], PhD; Saema Ansar[2*], PhD

[1] INSERM UMR-S U1237, Physiopathology and Imaging of Neurological Disorders, GIP Cyceron, Institut Blood and Brain @ Caen-Normandie (BB@C), Bd H. Becquerel, BP 5229, Caen, F-14074 France.

[2]Applied Neurovascular Research, Neurosurgery, Department of Clinical Sciences, Lund University, Lund, Sweden

[3]Experimental Vascular Research, Department of Clinical Sciences, Lund University, Lund, Sweden

[4]Department of Clinical Research, Caen-Normandie University Hospital, CHU, Caen, F-14000, France.

*Corresponding author
Saema Ansar, Applied Neurovascular Research, Neurosurgery, Department of Clinical Sciences, Klinikgatan 28, BMC C12, Lund University, SE-222 42 Lund, Sweden
Phone: +46 (0)70 3809786
E-mail: Saema.Ansar@med.lu.se

Figure 1.

a)

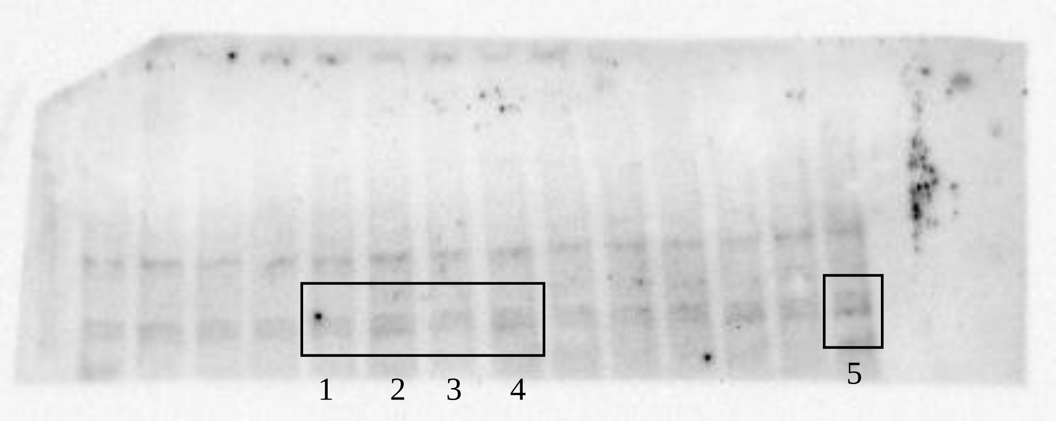

b)

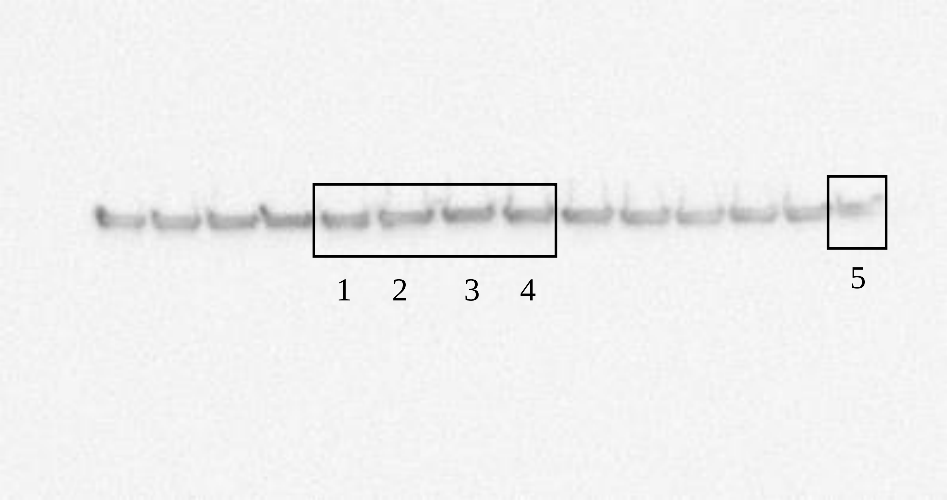

Figure 1. Complete western blot for representative picture of MMP-9 a) and its respective β-actin picture b) in Figure 4a in the article. 1 = Vehicle, 2 = rt-PA, 3 = rt-PA + U0126, 4 = U0126, 5 = control.

Figure 2

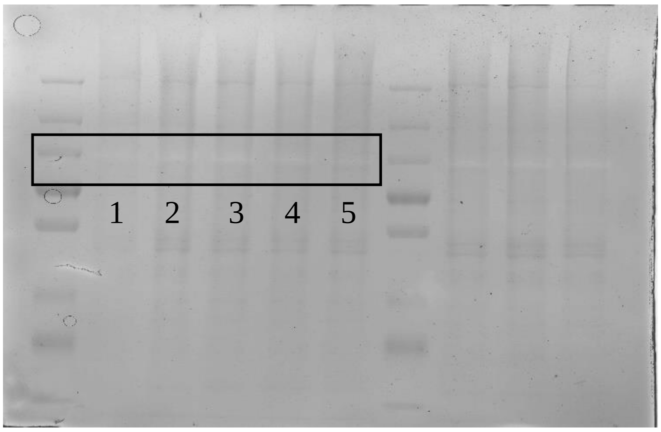

Figure 2. Complete zymography gel of representative image of MMP-9 in Figure 4b in the article. 1 = control, 2 = vehicle, 3 = rt-PA, 4 = rt-PA + U0126, 5 = U0126.

Figure 3.

a)

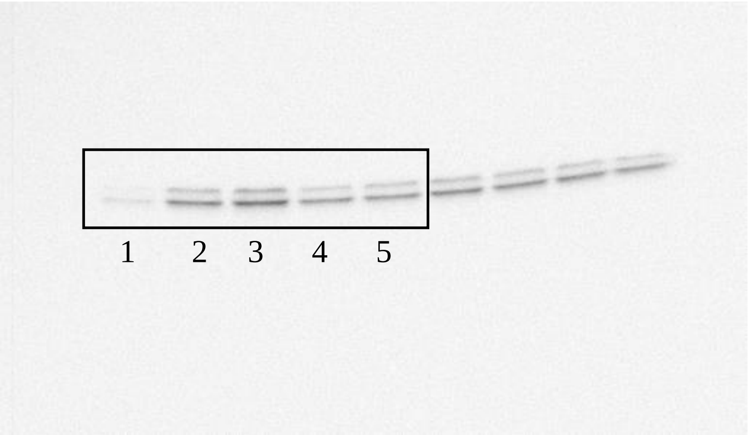

b)

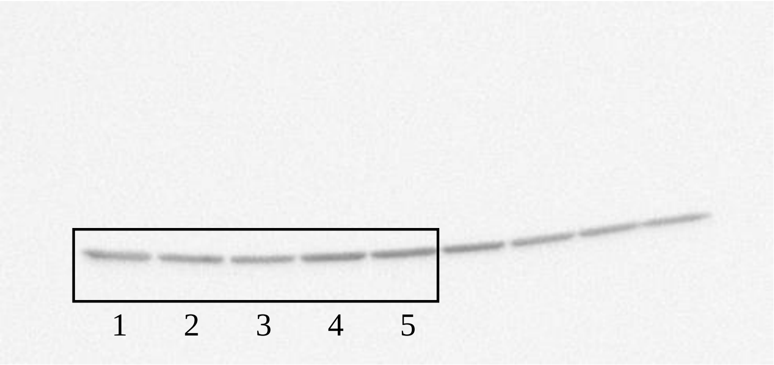

Figure 3. Complete western blot for representative picture of pERK1/2 a) and its respective β-actin picture b) in Figure 6a in the article. 1 = control, 2 = vehicle, 3 = rt-PA, 4 = rt-PA + U0126, 5 = U0126.
